# Acupuncture improves the emotion domain and lipid profiles in women with polycystic ovarian syndrome: a secondary analysis of a randomized clinical trial

**DOI:** 10.3389/fendo.2023.1237260

**Published:** 2023-08-29

**Authors:** Hui Chang, Baichao Shi, Hang Ge, Chengdong Liu, Lirong Wang, Chengcheng Ma, Lifeng Liu, Wanyu Zhang, Duojia Zhang, Yong Wang, Chi Chiu Wang, Xiaoke Wu

**Affiliations:** ^1^ Heilongjiang University of Chinese Medicine, Harbin, China; ^2^ First Affiliated Hospital, Heilongjiang University of Chinese Medicine, Harbin, China; ^3^ Affiliated Hospital of Jiangsu University, Zhenjiang, China; ^4^ Nanjing Universityg, Nanjin, China; ^5^ The Chinese University of Hong Kong, Hong Kong, Hong Kong, SAR, China; ^6^ Heilongjiang Provincial Hospital, Harbin, China

**Keywords:** polycystic ovarian syndrome, acupuncture, emotion, metabolism, ions

## Abstract

**Objective:**

This study aims to evaluate the effect of acupuncture on the emotion domain and metabolic parameters of Chinese women with polycystic ovarian syndrome (PCOS) by secondary analysis of a randomized clinical trial, conducted from 6 July 2012 to 7 October 2015.

**Method:**

In this study, we investigated the effects of acupuncture (458 patients) and sham acupuncture (468 patients) on metabolic parameters, serum ions, and all quality-of-life scale scores related to PCOS. The quality of life of patients was evaluated using five relevant scales, operated by the research assistant, namely, PCOSQ, SF-36, and ChiQOL, as well as Zung-SAS and Zung-SDS. Metabolic parameters and serum ions were measured.

**Results:**

A reduction in acne score, AN, Hcy, and LDL-C, and an increase in the level of lipoprotein α, Apo A1, and Apo A1/Apo B were observed in the acupuncture group after 4 months’ intervention after adjusting clomiphene and reproductive outcome (*p*< 0.05). An increase in SF-36 total scores, RP and RE scores, ChiQOL total scores, and emotion domain scores was observed in the acupuncture group after 4 months’ intervention, while PF and HT scores were decreased (adjusted *p*< 0.05). Those same changes were observed in sham acupuncture. Meanwhile, the serum levels of Ca, K, and Cl were elevated in the acupuncture group after the interventions (adjusted *p*< 0.005). There were no significant differences in HOMA-IR, MetS, FPG, FINS, HDL-C, TG, Apo B, and level of serum P, Mg, and Na. Also, no changes in BP, GH, VT, SF, physical form domain, and spirit domain were observed after treatment.

**Conclusion:**

Acupuncture can improve not only the emotional changes in SF-36 scores and ChiQOL scores, but also lipid metabolism, implying that it may have a correlation between emotional change and lipid metabolism. Furthermore, acupuncture can also regulate the changes of serum Ca, K, and Cl.

**Clinical trial registration:**

ClinicalTrials.gov, identifier NCT01573858.

## 1 Introduction

Polycystic ovarian syndrome (PCOS) is the most common endocrine disease in women of reproductive age, affecting more than 15%–20% of the population (1). Women with PCOS are frequently associated with metabolic abnormalities, including hyperinsulinemia, chronic low-grade inflammation, insulin resistance (IR), dyslipidemia, and obesity, and even anovulatory infertility and type 2 diabetes, as well as the risk of cardiovascular disease (CVD) (2). PCOS is not a simple physical disease; it is associated with mental and psychological disorders. It affects not only the health-related quality of life (HRQoL) of women, but also their psychological function (3). In most cases following a diagnosis of PCOS, there is an increased prevalence of depression and anxiety symptoms; perceived stress; self-reported medical diagnoses of depression, anxiety, or other major mental illnesses; and treatment for psychological conditions or mental illness (4–6). Compared with normal women, women with PCOS have significantly increased personality defects and psychiatric disorders, including anxiety, somatoform disorder and bipolar disorder, depression, delusional disorder and thought disorder, reduction of coping abilities and social skills, perceptual distortion and cognitive slippage, constant alertness and worry, chronic stress, and post-traumatic stress disorder, as well as inferiority, negativity, and social loneliness (7–9). Moreover, there is an overlap of clinical symptoms between the emotion domain and PCOS. There is a possibility of common associations between emotional disorders and PCOS-associated abnormalities including lipid metabolism. A previous study has shown that emotion, especially comorbidity among anxiety and depression, is connected with the metabolic syndrome (MetS), and the improvement in emotion may play an inhibitory role in the development of the syndrome, resulting in a reduced risk of MetS via physiological regulation systems (10), but equally, it has indicated the effect of metabolism on limbic function and emotional regulation, which can disrupt emotion regulation in the context of metabolic dysfunction (11).

Acupuncture is an integral part of traditional Chinese medicine (TCM), which dates back more than 3,000 years. In recent years, the use of acupuncture to regulate the disorder of reproductive endocrinology and infertility has become more popular worldwide (12). As an alternative therapy, acupuncture can improve the internal regulation function of the human body to prevent or treat diseases by receiving stimulation of different acupoints (13); in particular, efficacy on regulating menstruation and hormones is similar to recently clinical trials, reporting that acupuncture can induce regular menstrual cycles and ovulation, as well as decrease high circulating levels of luteinizing hormone and testosterone in women with PCOS (14) by affecting more functional signaling pathways associated with metabolism and hypothalamic pituitary ovarian axes (15). However, no efficacy on live birth rates by acupuncture was reported in PCOSAct of Wu’s study (16). Acupuncture had an effect of improving lipid profile, with an increase in levels of high-density lipoprotein cholesterol (HDL-C) and a decrease in other parameters including triglycerides (TG), total cholesterol (TC), and low-density lipoprotein cholesterol (LDL-C) (17). Previous clinical studies have demonstrated that acupuncture is effective for the improvement of quality of life and treatment of various diseases including anxiety disorders, fatigue, and depression (18, 19). Furthermore, acupuncture may alleviate anxiety during embryo transfer, and it has also been implied to have certain effects on perinatal depression (20). Although there are some studies on acupuncture treatment of glucose and lipid metabolism and emotional disorders in women with PCOS, the results have been conflicting.

The PCOSQ and SF-36 are most commonly used to assess the quality of life of women with PCOS. Among these, PCOSQ is a tool specifically designed and validated for evaluating HRQoL in PCOS, which together are able to capture different aspects of QoL in PCOS women and identify areas that can help improve QoL in these women (21). The Chinese QoL scale can provide effective information based on the universal QoL measures, and can also provide certain guidance for clinical applications (22). In addition, previous studies have demonstrated that acupuncture is effective in improving symptoms in PCOS with Self-assessed Anxiety Scale (SAS) and Self-assessed Depression Scale (SDS) scores (23). All the above scales have good applicability and reliability to evaluate the effectiveness of acupuncture in patients with PCOS. Therefore, this study, based on 1,000 patients with PCOS from the Acupuncture and Clomiphene in Polycystic Ovary Syndrome Trial (PCOSAct), is designed to explore the effects of acupuncture on metabolic parameters, serum ions, and the quality of life of PCOS.

## 2 Materials and methods

### 2.1 Design and target population

This is a *post-hoc* analysis of metabolic parameters, serum ions, and quality-of-life scales data from PCOSAct, a large-sample, multicenter, two-by-two factorial randomized controlled clinical trial collected at sites (27 hospitals) in mainland China between 6 July 2012 and 7 October 2015. In total, 1,000 participants were diagnosed with PCOS according to the modified Rotterdam criteria (24, 25): oligomenorrhea (defined as an intermenstrual interval >35 days and<8 menstrual bleedings in the past year) or amenorrhea (defined as an intermenstrual interval >90 days), together with clinical or biochemical hyperandrogenism (hirsutism determined by modified Ferriman-Gallwey hirsutism score ≥5 in Chinese) (26, 27) and/or polycystic ovaries (≥12 antral follicles 2–9 mm or ovarian volume ≥10 cm^3^). The study design, methods, and inclusion and exclusion criteria have been described in detail elsewhere (12), and the main results have been published (16). In brief, 926 women with PCOS were enrolled in the present study, and were assigned to the acupuncture group or the sham acupuncture group, with 458 patients and 468 patients per group, respectively.

The trial was registered on ClinicalTrials.gov (No. NCT01573858). The study was approved by the Regional Ethics Committee at The First Affiliated Hospital of Heilongjiang University of Traditional Chinese Medicine on 15 December 2011 (No. 2010HZYLL-010). All participants had signed informed consent before randomizing in the study.

### 2.2 Data collection

#### 2.2.1 Blood sampling

At baseline on day 3 of a spontaneous menstrual period or a withdrawal, bleeding was induced by medroxyprogesterone acetate in patients with irregular cycles without recent menses, and upon pregnancy, it was monitored weekly by urinary human chorionic gonadotropin test, or at the end of the 16 weeks of treatment, fasting blood samples were collected for measurement of metabolic parameters, lipid profile, and serum ions in those patients who are not pregnant at that time.

The anthropometric, degree of hirsutism and acne were collected and body mass index (BMI) was calculated as weight in kilograms divided by height in meters squared. All biochemical assays were carried out in a core laboratory after an overnight fast. All fasting blood collected at baseline and after intervention was used for metabolic parameters and serum ion assays. These were measured in samples stored at −80°C using an ELISA assay with no pre-dilution.

#### 2.2.2 Outcome measures

The biologic feature parameters of women with PCOS were obtained at baseline and at the follow-up visit, respectively, including age, male age, height, weight, BMI, waist, hip, waist–hip ratio (WHR), systolic pressure, diastolic pressure, respiration, pulse, Ferriman-Gallwey, acne scores, and acanthosis nigricans (AN).

In addition, metabolic parameters in blood test included homeostasis model assessment-insulin resistance (HOMA-IR), MetS, fasting plasma glucose (FPG), fasting plasma insulin (FINS), homocysteine (Hcy), HDL-C, LDL-C, lipoprotein α, TC, TG, Apolipoprotein A1 (Apo A1), Apolipoprotein B (Apo B), and Apo A1/Apo B, and serum ions including calcium (Ca), phosphorus (P), magnesium (Mg), potassium (K), sodium (Na), and chloride (Cl) were assayed.

Quality-of-life scales included Polycystic Ovary Syndrome Health-Related Quality of Life Questionnaire (PCOSQ, range of 1–7, with higher scores indicating better function) (28), Medical Outcomes Study 36-Item Short Form Health Survey (SF-36, range of 0–100, with higher scores indicating better function) (29), and Chinese Quality of Life Instrument (ChiQOL, range of 50–250, with higher scores indicating better function) (30), as well as the Zung-Self Rating Anxiety Scale (SAS, range of 25–100, with higher scores indicating worse anxiety) and Zung-Self Rating Depression Scale (SDS, range of 25–100, with higher scores indicating worse depression).

### 2.3 Statistical analyses

In the data description, categorical variables were summarized by frequencies. Continuous variables with normal distribution were presented as mean ± standard deviation ( $\bar{x}$ ± s). The data of non-normal distribution were analyzed by Mann–Whitney *U* test. Paired samples *t*-test or Mann–Whitney *U* test was used to analyze the continuous indices. SPSS 22.0 was used for analysis and *p*< 0.05 was considered to be statistically significant.

## 3 Results

### 3.1 Baseline characteristics

A total of 4,645 women with PCOS were screened from 6 July 2012 to 7 October 2015. A total of 1,000 eligible women were randomized and only 926 participants were included in the analysis. A total of 458 participants were assigned to the acupuncture group and 468 participants were assigned to the sham acupuncture group. There were no significant differences in baseline biological characteristics, metabolic parameters, and serum ions between two groups (**Table 1**). Quality-of-life scales including SAS, SDS, PCOSL, SF-36, and ChiQOL scores were paralleled between two groups at baseline (**Table 2**).

**Table 1:** Baseline characteristics of the subjects.

	Acupuncture group (*n* = 458)	Sham acupuncture group (*n* = 468)	Mean difference (95% CI)	*t*	*p*
Biological features					
Age	27.97 ± 3.33	27.87 ± 3.33	−0.11 (−0.52–0.31)	−0.51	0.6136
Male age	29.80 ± 4.03	29.81 ± 4.34	0.01 (−0.52–0.53)	0.02	0.9842
Height (cm)	161.24 ± 5.02	161.22 ± 5.16	−0.01 (−0.64–0.62)	−0.04	0.9695
Weight (kg)	62.53 ± 12.39	63.78 ± 12.45	1.25 (−0.29–2.79)	1.59	0.1115
BMI (kg/m^2^)	23.99 ± 4.29	24.47 ± 4.23	0.48 (−0.05–1.01)	1.79	0.0734
Waist (cm)	85.10 ± 11.61	85.75 ± 11.35	0.65 (−0.78–2.07)	0.89	0.3722
Hip (cm)	98.25 ± 8.49	98.65 ± 8.76	0.40 (−0.67–1.48)	0.74	0.4598
WHR	0.86 ± 0.07	0.87 ± 0.07	0.00 (−0.01–0.01)	−0.84	0.4006
Systolic pressure (mmHg)	112.00 ± 9.33	112.61 ± 9.49	0.61 (−0.55–1.78)	1.03	0.3026
Diastolic pressure (mmHg)	74.80 ± 7.86	74.91 ± 7.93	0.11 (−0.87–1.09)	0.22	0.8233
Respiration (beats/min)	18.09 ± 1.69	18.01 ± 1.51	−0.08 (−0.28–0.12)	−0.83	0.409
Pulse (beats/min)	75.91 ± 6.33	76.22 ± 6.15	0.31 (−0.47–1.08)	0.78	0.4373
Ferriman-Gallwey	3.14 ± 2.85	2.93 ± 2.76	−0.22 (−0.57–0.13)	−1.23	0.2189
Acne sore	1.76 ± 0.73	1.71 ± 0.64	−0.05 (−0.14–0.04)	−1.1	0.2724
Acanthosis nigricans	1.73 ± 0.61	1.73 ± 0.68	−0.00 (−0.09–0.08)	−0.09	0.9274
Metabolic parameters					
HOMA-IR	23.82 ± 28.76	21.68 ± 21.29	−2.14 (−5.37–1.08)	−1.30	0.1926
Metabolic syndrome	71 (14.2)	83 (16.6)	-	-	0.2931
FPG (mmol/L)	5.07 ± 0.93	5.01 ± 1.04	−0.06 (−0.19–0.06)	−0.95	0.3408
FINS (pmol/L)	99.18 ± 96.88	92.97 ± 78.74	−6.21 (−17.41–4.99)	−1.09	0.277
Hcy (μmol/L)	8.39 ± 5.07	8.29 ± 4.64	−0.10 (−0.72–0.53)	−0.31	0.7574
HDL-C (mmol/L)	1.29 ± 0.36	1.26 ± 0.38	−0.03 (−0.08–0.02)	−1.32	0.1861
LDL-C (mmol/L)	3.00 ± 0.89	2.93 ± 0.86	−0.06 (−0.18–0.05)	−1.15	0.2511
Lipoprotein (mg/L)	132.65 ± 101.58	126.32 ± 97.97	−6.34 (−19.01–6.34)	−0.98	0.3268
Total cholesterol (mmol/L)	4.80 ± 1.10	4.68 ± 1.07	−0.11 (−0.25–0.02)	−1.62	0.105
Triglyceride (mmol/L)	1.59 ± 0.95	1.55 ± 0.87	−0.03 (−0.15–0.08)	−0.58	0.559
Apo A1(g/L)	1.53 ± 0.31	1.49 ± 0.32	−0.04 (−0.08 to −0.00)	−1.99	0.0474
Apo B (g/L)	0.91 ± 0.29	0.89 ± 0.28	−0.02 (−0.06–0.02)	−1.11	0.2669
Serum ion test					
Ca (mmol/L)	1.95 ± 0.43	1.96 ± 0.39	0.02 (−0.03–0.07)	0.68	0.4978
P (mmol/L)	1.13 ± 0.26	1.15 ± 0.34	0.02 (−0.02–0.06)	0.9	0.3666
Mg (mmol/L)	0.86 ± 0.18	0.87 ± 0.15	0.00 (−0.02–0.03)	0.44	0.6568
K (mmol/L)	4.14 ± 0.92	4.23 ± 0.88	0.09 (−0.03–0.20)	1.5	0.1329
Na (mmol/L)	135.66 ± 16.12	136.53 ± 14.21	0.87 (−1.08–2.81)	0.88	0.3815
Cl (mmol/L)	93.39 ± 12.24	94.08 ± 10.86	0.69 (−0.79–2.16)	0.91	0.3631

Data are median (IQR).

p-value represents comparison between acupuncture and sham acupuncture groups before treatment.

**Table 2:** The quality of life outcomes sore at baseline.

	Acupuncture group (*n* = 458)	Sham acupuncture group (*n* = 468)	Mean difference (95% CI)	*t*	P
SAS score	33.71 ± 6.93	33.86 ± 6.82	0.15 (−0.70–1.01)	0.35	0.7245
SDS score	35.24 ± 8.61	35.48 ± 8.21	0.24 (−0.81–1.29)	0.45	0.6525
**PCOSQOL score**	127.88 ± 28.38	127.09 ± 29.47	−0.79 (−4.38–2.81)	−0.43	0.6672
Emotions	4.27 ± 1.11	4.31 ± 1.14	0.04 (−0.10–0.18)	0.56	0.5777
Body hair	5.31 ± 1.52	5.25 ± 1.61	−0.06 (−0.26–0.13)	−0.63	0.5276
Weight	4.29 ± 1.72	4.20 ± 1.69	−0.08 (−0.29–0.13)	−0.75	0.4515
Infertility	3.65 ± 1.35	3.64 ± 1.41	−0.01 (−0.18–0.16)	−0.08	0.9368
Menstrual problems	4.41 ± 1.10	4.35 ± 1.14	−0.06 (−0.20–0.08)	−0.9	0.3666
**SF**-**36 score**	104.15 ± 4.52	104.06 ± 4.54	−0.09 (−0.65–0.47)	−0.32	0.7518
Physical functioning	93.36 ± 9.70	93.29 ± 9.53	−0.07 (−1.26–1.13)	−0.11	0.9126
Role-physical	23.35 ± 29.99	21.49 ± 30.55	−1.86 (−5.62–1.90)	−0.97	0.3321
Bodily pain	77.64 ± 12.96	76.75 ± 13.68	−0.89 (−2.54–0.77)	−1.05	0.2933
General health	52.91 ± 11.64	53.39 ± 12.20	0.48 (−1.01–1.96)	0.63	0.5288
Vitality	42.73 ± 11.92	42.20 ± 12.36	−0.53 (−2.04–0.98)	−0.69	0.4897
Social functioning	49.00 ± 13.09	48.95 ± 10.97	−0.05 (−1.55–1.45)	−0.07	0.9435
Role-emotional	26.40 ± 34.62	24.23 ± 34.90	−2.17 (−6.49–2.15)	−0.99	0.3244
Mental health	60.02 ± 10.46	60.84 ± 9.89	0.83 (−0.44–2.09)	1.28	0.1997
Health transition	48.55 ± 25.80	47.29 ± 25.62	−1.26 (−4.46–1.93)	−0.77	0.4388
**ChiQOL score**	148.20 ± 11.04	148.22 ± 11.28	0.03 (−1.36–1.42)	0.04	0.9674
Physical form domain	3.35 ± 0.80	3.25 ± 0.85	−0.10 (−0.20–0.00)	−1.93	0.0536
Spirit domain	95.53 ± 8.36	95.58 ± 8.21	0.05 (−0.98–1.08)	0.1	0.9176
Emotion domain	49.32 ± 7.04	49.39 ± 7.03	0.08 (−0.80–0.95)	0.17	0.8654

Data are median (IQR).

p-value represents comparison between acupuncture and sham acupuncture groups before treatment.

### 3.2 Biological features

Acne scores and AN of biological features were decreased after acupuncture intervention in both groups after adjusting clomiphene and reproductive outcome (*p*< 0.001). Also, there was a significant decrease in acne scores in the acupuncture group after intervention, compared with the sham acupuncture group (adjusted *p* = 0.0102). By contrast, no significant difference in weight, BMI, waist, hip, WHR, systolic pressure, diastolic pressure, respiration, pulse, and Ferriman-Gallwey were found when comparing pre-treatment and post-treatment or between acupuncture and sham acupuncture (**Table 3**).

**Table 3:** Metabolic parameters and serum ion after acupuncture and sham acupuncture.

	Acupuncture group				Sham acupuncture group					
	Pre-treatment (*n* = 458)	Post-treatment (*n* = 458)	*p*	*p*	Pre-treatment (*n* = 468)	Post-treatment (*n* = 468)	*p*	^a^ *p*	*p*1	^a^ *p*1
Biological features										
Weight (kg)	62.53 ± 12.39	62.26 ± 12.39	0.7382	0.7895	63.78 ± 12.45	63.52 ± 11.98	0.7410	0.7683	0.9118	0.9011
BMI (kg/m^2^)	23.99 ± 4.29	23.90 ± 4.29	0.7424	0.7915	24.47 ± 4.23	24.39 ± 4.10	0.7488	0.7782	0.8839	0.8954
Waist (cm)	85.10 ± 11.61	84.85 ± 11.54	0.7391	0.7677	85.75 ± 11.35	85.39 ± 11.09	0.6134	0.6378	0.9752	0.9761
Hip (cm)	98.25 ± 8.49	98.15 ± 8.58	0.8607	0.8814	98.65 ± 8.76	98.45 ± 8.58	0.7115	0.7279	0.8975	0.8912
WHR	161.24 ± 5.02	161.24 ± 5.08	0.9887	0.7548	0.87 ± 0.07	0.87 ± 0.07	0.7101	0.7328	0.8666	0.8638
Systolic pressure (mmHg)	112.00 ± 9.33	112.07 ± 8.59	0.9024	0.8631	112.61 ± 9.49	112.48 ± 8.51	0.8248	0.8321	0.486	0.4861
Diastolic pressure (mmHg)	74.80 ± 7.86	74.95 ± 7.54	0.7725	0.7577	74.91 ± 7.93	75.48 ± 7.73	0.2598	0.2525	0.3863	0.3822
Respiration (beats/min)	18.09 ± 1.69	17.96 ± 1.72	0.2204	0.2308	18.01 ± 1.51	18.02 ± 1.76	0.8988	0.9181	0.109	0.1162
Pulse (beats/min)	75.91 ± 6.33	76.03 ± 6.39	0.7769	0.7647	76.22 ± 6.15	76.20 ± 5.94	0.9668	0.9624	0.8016	0.8114
Ferriman-Gallwey	3.14 ± 2.85	2.81 ± 2.70	0.0639	0.0670	2.93 ± 2.76	2.67 ± 2.66	0.1420	0.1465	0.5203	0.5105
Acne score	0.70 ± 0.46	0.24 ± 0.43	<0.0001	<0.0001	0.65 ± 0.48	0.27 ± 0.44	<0.0001	<0.0001	0.0096	0.0102
Acanthosis nigricans	1.21 ± 0.47	0.18 ± 0.44	<0.0001	<0.0001	1.21 ± 0.48	0.19 ± 0.44	<0.0001	<0.0001	0.81	0.8395
Metabolic parameters										
HOMA-IR	23.82 ± 28.76	26.24 ± 33.12	0.2381	0.2310	21.68 ± 21.29	23.28 ± 22.70	0.2706	0.2659	0.8554	0.8736
Metabolic syndrome	71(14.2)	57(11.4)	0.1851	0.1831	83(16.6)	68(13.6)	0.1852	0.1840	-	-
FPG (mmol/L)	5.07 ± 0.93	5.07 ± 1.27	0.9712	0.9980	5.01 ± 1.04	4.99 ± 1.19	0.7457	0.6999	0.5941	0.5654
FINS (pmol/L)	99.18 ± 96.88	107.25 ± 105.13	0.2243	0.2199	92.97 ± 78.74	100.59 ± 86.67	0.1609	0.1565	0.8581	0.8393
Hcy (μmol/L)	8.39 ± 5.07	6.76 ± 4.29	<0.0001	<0.0001	8.29 ± 4.64	6.26 ± 3.69	<0.0001	<0.0001	0.1128	0.0972
HDL-C (mmol/L)	1.29 ± 0.36	1.33 ± 0.41	0.1140	0.1175	1.26 ± 0.38	1.29 ± 0.39	0.2772	0.2872	0.6753	0.6600
LDL-C (mmol/L)	3.00 ± 0.89	2.86 ± 1.06	0.0346	0.0365	2.93 ± 0.86	2.75 ± 0.91	0.0012	0.0012	0.6249	0.5895
Lipoprotein α(mg/L)	132.65 ± 101.58	187.75 ± 111.97	<0.0001	<0.0001	126.32 ± 97.97	176.7 ± 113.90	<0.0001	<0.0001	0.4808	0.5429
Total cholesterol (mmol/L)	4.80 ± 1.10	4.65 ± 1.28	0.0610	0.0691	4.68 ± 1.07	4.51 ± 1.16	0.0213	0.0204	0.9273	0.8891
Triglyceride (mmol/L)	1.59 ± 0.95	1.49 ± 0.85	0.0969	0.1055	1.55 ± 0.87	1.56 ± 0.98	0.8853	0.8612	0.046	0.0476
Apolipoprotein A1(g/L)	1.53 ± 0.31	1.57 ± 0.38	0.0382	0.0403	1.49 ± 0.32	1.54 ± 0.36	0.0202	0.0223	0.63	0.6351
Apolipoprotein B (g/L)	0.91 ± 0.29	0.88 ± 0.33	0.2024	0.2166	0.89 ± 0.28	0.86 ± 0.29	0.1231	0.1269	0.8685	0.8823
ApoA1/ApoB	1.84 ± 0.70	1.99 ± 0.77	0.0023	0.0024	1.80 ± 0.59	1.94 ± 0.66	0.0007	0.0008	-	-
Serum ion test										
Ca (mmol/L)	1.95 ± 0.43	2.04 ± 0.55	0.0026	0.0025	1.96 ± 0.39	2.02 ± 0.55	0.0703	0.0608	0.2101	0.1942
P (mmol/L)	1.13 ± 0.26	1.11 ± 0.30	0.4308	0.4418	1.15 ± 0.34	1.13 ± 0.38	0.5444	0.6009	0.8068	0.7768
Mg (mmol/L)	0.86 ± 0.18	0.88 ± 0.21	0.2758	0.2735	0.87 ± 0.15	0.87 ± 0.21	0.7689	0.7219	0.3738	0.3536
K (mmol/L)	4.14 ± 0.92	4.44 ± 1.97	0.0032	0.0032	4.23 ± 0.88	4.32 ± 1.73	0.3369	0.3435	0.0516	0.0502
Na (mmol/L)	135.66 ± 16.12	136.96 ± 22.03	0.3085	0.3129	136.53 ± 14.21	136.61 ± 22.21	0.9485	0.9258	0.233	0.2180
Cl (mmol/L)	93.39 ± 12.24	97.88 ± 17.92	<0.0001	<0.0001	94.08 ± 10.86	97.60 ± 18.04	0.0003	0.0003	0.2471	0.2324

Data are median (IQR).

p-values represent comparison before and after treatment within acupuncture and sham acupuncture groups.

a p-values represent comparison before and after treatment within acupuncture and sham acupuncture groups after adjusting clomiphene and reproductive outcome.

p1-values represent comparison of the difference value before and after treatment between acupuncture and sham acupuncture groups.

a p1-values represent comparison of the difference value before and after treatment between acupuncture and sham acupuncture groups after adjusting clomiphene and reproductive outcome.

### 3.3 Metabolic parameters

The level of serum Hcy was significantly decreased after treatment in both groups (adjusted *p*< 0.001). Additionally, the levels of LDL-C and Apo A1 were significantly decreased, while the serum levels of lipoprotein α, Apo A1/Apo B, and TC were significantly increased after acupuncture treatment (adjusted *p*< 0.05). By contrast, no significant differences in HOMA-IR, MetS, FPG, FINS, Hcy, HDL-C, LDL-C, lipoprotein α, TC, Apo A1, and Apo B (except for TG) were found when comparing pre-treatment and post-treatment or between acupuncture and sham acupuncture (adjusted *p* = 0.0476) (**Table 3**).

### 3.4 Serum ions

The levels of serum Cl, serum Ca, and serum K were both significantly increased in the acupuncture group after intervention of 4 months compared with pre-treatment (adjusted *p*< 0.05). Also, the levels of Cl were increased in the sham acupuncture group. There were no significant differences in P, Mg, and Na when comparing pre-treatment and post-treatment or between acupuncture and sham acupuncture (**Table 3**).

### 3.5 The quality-of-life scales

The total scores, role-physical (RP) scores, and role-emotional (RE) scores of SF-36 were significantly increased after 4 months’ acupuncture intervention (adjusted *p*< 0.05),but equally, the total score and emotion domain scores of ChiQOL scale were also significantly increased. By contrast, the scores on physical functioning (PF) and health transition (HT) of SF-36 were significantly decreased after intervention (adjusted *p*< 0.05). Those same changes were found in the sham acupuncture group.

There were no significant differences in bodily pain (BP), general health (GH), vitality (VT), social functioning (SF), RE, mental health (MH), and HT scores of SF-36 and total scores, emotion, body hair, weight, infertility problems of PCOSQOL, Physical Form Domain, and Spirit Domain of ChiQOL scores when comparing pre-treatment and post-treatment or between acupuncture and sham acupuncture, as well as SAS and SDS scores (**Table 4**).

**Table 4:** Quality of life outcomes score from pre-treatment to post-treatment.

	Acupuncture group				Sham acupuncture group					
	Pre-treatment (*n* = 458)	Post-treatment (*n* = 458)	*p*	^a^ *p*	Pre-treatment (*n* = 468)	Post-treatment (*n* = 468)	*p*	^a^ *p*	*p*1	^a^ *p*1
SAS score	33.71 ± 6.93	34.50 ± 7.35	0.0840	0.0780	33.86 ± 6.82	34.20 ± 7.13	0.4532	0.4610	0.1727	0.1734
SDS score	35.24 ± 8.61	36.13 ± 8.61	0.1095	0.1023	35.48 ± 8.21	36.09 ± 8.40	0.2570	0.2620	0.3955	0.4055
**PCOSQOL score**	127.88 ± 28.38	128.71 ± 28.08	0.6488	0.6907	127.09 ± 29.47	127.05 ± 28.94	0.9834	0.9521	0.7371	0.7532
Emotions	4.27 ± 1.11	4.25 ± 1.10	0.8180	0.7721	4.31 ± 1.14	4.24 ± 1.08	0.3498	0.3255	0.4056	0.4030
Body hair	5.31 ± 1.52	5.27 ± 1.52	0.6960	0.7000	5.25 ± 1.61	5.21 ± 1.51	0.6924	0.6721	0.735	0.7378
Weight	4.29 ± 1.72	4.31 ± 1.64	0.8319	0.8780	4.20 ± 1.69	4.23 ± 1.66	0.8295	0.8744	0.588	0.5646
Infertility	3.65 ± 1.35	3.73 ± 1.30	0.3455	0.3746	3.64 ± 1.41	3.63 ± 1.43	0.9385	0.9116	0.1827	0.1866
Menstrual problems	4.41 ± 1.10	4.41 ± 1.12	0.9750	0.9552	4.35 ± 1.14	4.32 ± 1.15	0.7299	0.7449	0.7393	0.7563
**SF**-**36 score**	104.15 ± 4.52	106.89 ± 5.78	<0.0001	<0.0001	104.06 ± 4.54	106.78 ± 5.93	<0.0001	<0.0001	0.8016	0.7700
Physical functioning	93.36 ± 9.70	91.65 ± 11.53	0.0125	0.0129	93.29 ± 9.53	91.31 ± 11.43	0.0034	0.0034	0.9231	0.9268
Role-physical	23.35 ± 29.99	71.48 ± 32.39	<0.0001	<0.0001	21.49 ± 30.55	74.04 ± 32.23	<0.0001	<0.0001	0.1552	0.1594
Bodily pain	77.64 ± 12.96	76.78 ± 13.21	0.3105	0.2987	76.75 ± 13.68	75.80 ± 13.80	0.2821	0.3107	0.95	0.9026
General health	52.91 ± 11.64	53.60 ± 11.56	0.3558	0.3726	53.39 ± 12.20	54.00 ± 11.86	0.4276	0.4243	0.8012	0.8245
Vitality	42.73 ± 11.92	43.93 ± 11.97	0.1203	0.1213	42.20 ± 12.36	42.91 ± 12.21	0.3652	0.3451	0.5652	0.5629
Social functioning	49.00 ± 13.09	49.49 ± 11.79	0.5464	0.5464	48.95 ± 10.97	49.41 ± 12.15	0.5280	0.4826	0.8944	0.8264
Role-emotional	26.40 ± 34.62	71.43 ± 36.43	<0.0001	<0.0001	24.23 ± 34.90	73.40 ± 36.16	<0.0001	<0.0001	0.2813	0.2930
Mental health	60.02 ± 10.46	58.87 ± 10.56	0.0902	0.0875	60.84 ± 9.89	59.35 ± 10.83	0.0255	0.0247	0.8506	0.8769
Health transition	48.55 ± 25.80	42.48 ± 26.33	0.0003	0.0003	47.29 ± 25.62	39.89 ± 26.15	<0.0001	<0.0001	0.7356	0.7150
**ChiQOL score**	148.20 ± 11.04	149.80 ± 11.12	0.0254	0.0268	148.22 ± 11.28	149.78 ± 12.32	0.0410	0.0391	0.6637	0.6693
Physical form domain	3.35 ± 0.80	3.34 ± 0.82	0.9104	0.8837	3.25 ± 0.85	3.27 ± 0.80	0.6912	0.7051	0.5796	0.5996
Spirit domain	95.53 ± 8.36	96.34 ± 8.48	0.1386	0.1435	95.58 ± 8.21	96.18 ± 8.86	0.2791	0.2620	0.3763	0.3953
Emotion domain	49.32 ± 7.04	51.12 ± 7.36	0.0350	0.0353	49.39 ± 7.03	50.33 ± 7.44	0.0445	0.0451	0.8141	0.8332

Data are median (IQR).

p-values represent comparison before and after treatment within acupuncture and sham acupuncture groups.

a p-values represent comparison before and after treatment within acupuncture and sham acupuncture groups after adjusting clomiphene and reproductive outcome.

p1-values represent comparison of the difference value before and after treatment between acupuncture and sham acupuncture groups.

b p1-values represent comparison of the difference value before and after treatment between acupuncture and sham acupuncture groups after adjusting clomiphene and reproductive outcome.

## 4 Discussion

Our results showed that total scores and RP and RE scores in the SF-36 scale after treatment in the two groups were significantly higher than before treatment, whereas PF, MH, and HT scores were significantly lower than those before treatment, after adjusting clomiphene and reproductive outcome. In the ChiQOL scale, the significant increase in total scores and emotion domain scores was observed in the two groups compared with pre-treatment. These findings indicated that acupuncture could improve the emotion domain of PCOS patients, and the same effects were found in sham acupuncture. The SF-36 questionnaire-a is a concise health survey that comprehensively summarizes the survival quality of respondents; it contains 36 items divided into nine areas of HRQoL. Our study revealed that acupuncture significantly improved the scores of RP and RE in SF-36 of patients with PCOS, which were decreased after acupuncture treatment in previous research (3). The ChiQOL questionnaire is a new universal scale based on TCM (30, 31). The total scores and emotion domain scores in the ChiQOL questionnaire were significantly increased after acupuncture intervention, which indicated that acupuncture could improve the quality of life of Chinese people; Dong et al.’s study had the same results (3). Some research reported that acupuncture played a significant improvement in the physical form domain and a significantly and continuously improved trend in the vitality domain (32), but in our study, the scores of PF, MH, and HT in SF-36 were decreased after acupuncture intervention, which may be related to the physical and mental health damage caused by long-term infertility of PCOS and could not be corrected by acupuncture. Moreover, sham acupuncture was not completely useless; the same effects were found in sham acupuncture group. The PCOSQ as the only validated disease-specific questionnaire has been used to evaluate the HRQoL of women with PCOS currently, which has been administered to understand women experiences of emotions, body hair, weight, infertility, and menstrual problems of PCOS symptomatology in detail (28, 33). It has been revealed that acupuncture plays a significant role in ameliorating negative emotion in PCOS patients, which was decreased in SAS and SDS scores and increased in PCOSQ scores (23), but no differences in PCOSQ, SAS, and SDS scores were observed in our study.

The level of serum Hcy was significantly decreased after treatment in both groups (adjusted *p*< 0.001). Additionally, the levels of LDL-C and Apo A1 were significantly decreased, while the serum levels of lipoprotein α, Apo A1/Apo B, and TC were significantly increased after acupuncture treatment (adjusted *p*< 0.05). It is a controversial issue whether acupuncture can improve lipid metabolism in patients with PCOS. A previous study indicated that acupuncture could improve TG levels in lipid metabolism of PCOS (34), whereas acupuncture had no effect on LDL-C, TG, and TC in lipid metabolism, as illustrated in another study (35). Our study found that the TG in lipid metabolism indicators was significantly different between two groups after treatment. Our findings showed that acupuncture was closely associated with a decrease in the level of LDL-C and an increase in the level of lipoprotein α and Apo A1, and slightly increased the ratio of Apo A1/Apo B in lipid profiles in patients with PCOS, while the sham acupuncture group could increase the TC level. Lai et al.’s clinical trials reported that the LDL-C level was reduced by acupuncture treatment (36); the same results have been found in our study. However, no significant change in HDL-C levels after acupuncture treatment was reported in our study, and the finding was not consistent with another study (37). Furthermore, acupuncture could significantly improve the acne symptoms of patients with PCOS, while other biological features such as BMI, WHR, and AN were not improved, compared with the sham acupuncture group. However, no significant differences in HOMA-IR, MetS, FPG, FINS, Hcy, HDL-C, LDL-C, lipoprotein α, TC, Apo A1, and Apo B (except for TG) were found when comparing pre-treatment and post-treatment or between acupuncture and sham acupuncture (adjusted *p* = 0.0476).

Our research found that acupuncture can regulate emotion and improve abnormal lipid metabolism and ion disorders, and these may have a certain relationship. Poor mental health was significantly associated with elevated levels of TG and LDL-C. A recent cross-sectional study demonstrated that female participants who experienced high stress showed higher levels of LDL-C compared with the low-stress participants, suggesting that higher levels of blood lipids and lipoprotein α were related with psychological stress and emotional disorders (38). The same was found in patients with non-alcoholic fatty liver disease (NAFLD). There were impaired health status in NAFLD patients, including physical, emotional, mental, and social wellbeing (39), which also presented a complex, bidirectional relationship with lipid metabolism (40). As acupuncture could reduce the accumulation of intra-abdominal fat, inhibit lipid absorption in the small intestine, and downregulate the level of blood lipid, acupuncture may have potentially variable beneficial effects on improving the lipid metabolism in the presence of abnormal liver metabolism (41). These studies have suggested that there is a relationship between lipid metabolism and emotional domain. Our findings indicated that the role of acupuncture in improving emotion domain seemed to be related to the improvement of metabolic disorders in women with PCOS. Acupuncture treatment was found to positively improve leptin sensitivity in brain, thereby altering hepatic lipid metabolism and reducing depression-like behavior in chronic restraint stress mice (42). Clinical studies and lines of evidence from laboratory indicated that acupuncture regulates the endocrine system and modulates relevant molecules of metabolism in patients of simple obesity (43). As for the detection of dyslipidemia in both PCOS-like and obese rats, representative indices such as TG, TC, LDL-C, and Apo E were increased, while HDL-C was decreased. Moreover, there was also a certain degree of liver dysfunction, and all these abnormal changes could be reverted or improved with electroacupuncture (44). Therefore, the improvement of PCOS may represent a positive step towards relieving mental symptoms and preventing mental complications by improving lipid metabolism. Moreover, in the perspective of TCM theory, the liver governs emotions, while the main organ of lipid metabolism is the liver.

We found that the changes of emotion and lipid provided molecular biological evidence for the scientific connotation of the theory of liver governing emotion in TCM. A previous study also found that acupuncture could effectively modify hepatic lipid metabolism by increasing the sensitivity to leptin. This is consistent with the theory of liver master sentiment in TCM (45). We also found that the effect of acupuncture on emotion and lipid metabolism played a role in sham acupuncture, probably due to the physiological effects of shallow acupuncture by stimulating the skin and causing afferent nerve activity, which, in turn, affects the corresponding functional areas of the brain, producing a “limbic contact response” (46). In addition, the patient’s cognition of the acupuncturist; the patient’s knowledge, attitudes, and behaviors; the patient’s relationship with acupuncturist; and the trial environment increased the non-specific effects of acupuncture and sham acupuncture by prompting patients to change their healthy lifestyle (47).

Additionally, we found that the levels of serum Cl, serum Ca, and serum K have been both significantly increased in the acupuncture group after an intervention of 4 months compared with pre-treatment (adjusted *p*< 0.05). Also, the level of Cl was increased in the sham acupuncture group. It was reported that acupuncture could change the concentration of Ca^2+^ on the meridian line, which was one of the key factors of acupuncture effect, and the relationship between Ca^2+^ and acupuncture effect implied that Ca^2+^ was closely related to the neuroendocrine–immune network at acupoint (48). Studies on the mechanism of acupuncture seemed to suggest that the direct consequence in connection to acupuncture was the necessity of increasing intracellular Ca^2+^ (49). Since acupuncture was capable of delivering the extra Ca^2+^, the improvement of ions in patients with PCOS seems to coincide with its ability.

In our study, a decrease in Hcy levels after acupuncture intervention was of novel interest. Studies have illustrated that elevated concentration of Hcy can affect blood vessels, which may have contributed to the deterioration of atherosclerosis and endothelial dysfunction by damaging the blood vessel, thus increasing the risk of CVD (50). Hcy may play a role in the long-term complications of PCOS, especially the arterial stiffness of CVD (51), but it is not clear whether it is connected with the metabolism of PCOS. It is well known that the level of Hcy is elevated in women with PCOS. A meta-analysis has shown that the Hcy level in obese patients with PCOS is significantly higher than that in nonobese patients (52), which showed that Hcy seems to be related to fat metabolism. Additionally, hyperhomocysteinemia contributes to elevated pregnancy loss and reduced ovulation in PCOS, indicating that the higher Hcy level leads to reproductive defects (53). Our study also found that acupuncture could reduce Hcy levels, but unfortunately did not have an effect on the live birth rate (16), which was worthy of further study in the future. Nevertheless, no significant differences in HOMA-IR, MetS, FPG, and FINS were found when comparing pre-treatment and post-treatment or between acupuncture and sham acupuncture, which was consistent with Dong’s report (35).

### 4.1 Strengths and limitations

This research is the first study to use the most comprehensive scale to evaluate the effect of acupuncture on women with PCOS, and it is found that acupuncture can regulate emotion and lipid metabolism disorder, which may have a correlation, based on 1,000 PCOS women from randomized controlled trials that include the factorial design, adequate power, similar withdrawal rates among groups, and high adherence to treatment. Furthermore, acupuncture had a positive effect on levels of serum Ca, serum K, and serum Cl, which may also be another mechanism of acupuncture regulating emotion.

However, there were some limitations in our study. Firstly, both acupuncture and sham acupuncture played a role in the present study, and it was not clear what the specific mechanism was. On the one hand, sham acupuncture may have a comforting needle effect, and on the other hand, the techniques of acupuncturists may be different, although our acupuncturists had undergone several rounds of standardized and uniform training. Secondly, there was subjectivity in the assessment of acupuncture scale by PCOS patients. Although our research assistants have undergone intensive training on a regular basis, the particular variations in individual indicators may be caused by the subjective reasons of the research assistants. Thirdly, the subgroup analysis was not carried out based on BMI, which may lead to the deviation of the research results. Finally, the article only determined the potential relationships, and the specific mechanisms of the regulatory effect of acupuncture on emotion and lipid metabolism need to be further investigated.

## 5 Conclusions

Acupuncture can improve not only the emotional changes in SF-36 scale scores and ChiQOL scale scores, but also lipid metabolism. There is a correlation between emotional change and lipid metabolism. Meanwhile, acupuncture can regulate the changes of serum Ca, K, and Cl ions, which may be another mechanism of acupuncture in the treatment of PCOS. Moreover, sham acupuncture may not be completely ineffective, which may be related to the improvement in some aspects of patients with PCOS through the placebo effect.

## Data availability statement

The original contributions presented in the study are included in the article/supplementary material, and further inquiries can be directed to the corresponding author/s.

## Ethics statement

The studies involving humans were approved by The Regional Ethics Committee at The First Affiliated Hospital of Heilongjiang University of Traditional Chinese Medicine on December 15, 2011 (No: 2010HZYLL-010). The studies were conducted in accordance with the local legislation and institutional requirements. The participants provided their written informed consent to participate in this study. Inclusion of identifiable human data. Written informed consent was obtained from the individual(s) for the publication of any potentially identifiable images or data included in this article.

## Author contributions

HC, CW, and XW initiated, designed, and supervised the study. HC, LW, CM, LL, WZ, and HG analyzed the data. HC, BS, and DZ drafted the manuscript. YW, CW, and XW revised the manuscript. All authors have read and approved the final version of the manuscript accepted for publication. All authors accept responsibility for the accuracy and integrity of all aspects of the study.

## References

[1] Moulana M. Androgen-induced cardiovascular risk in polycystic ovary syndrome: the role of T lymphocytes. Life (2023) 13:1010. doi: 10.3390/life13041010 37109539 PMC10145997

[2] Dapas M Dunaif A. Deconstructing a syndrome: genomic insights into PCOS causal mechanisms and classification. Endocrine Rev (2022) 43:927–65. doi: 10.1210/endrev/bnac001 PMC9695127 35026001

[3] Wang Z Dong H Wang Q Zhang L Wu X Zhou Z. Effects of electroacupuncture on anxiety and depression in unmarried patients with polycystic ovarian syndrome: secondary analysis of a pilot randomised controlled trial. Acupuncture Med (2019) 37:40–6. doi: 10.1136/acupmed-2017-011615 30843421

[4] Karjula S Morin-Papunen L Auvinen J Ruokonen A Puukka K Franks S. Psychological distress is more prevalent in fertile age and premenopausal women with PCOS symptoms: 15-year follow-up. J Clin Endocrinol Metab (2017) 102:1861–9. doi: 10.1210/jc.2016-3863 PMC5470769 28323926

[5] Damone AL Joham AE Loxton D Earnest A Teede HJ Moran LJ. Depression, anxiety and perceived stress in women with and without PCOS: a community-based study. psychol Med (2019) 49:1510–20. doi: 10.1017/S0033291718002076 30131078

[6] Hasan M Sultana S Sohan M Parvin S Rahman MA Hossain MJ. Prevalence and associated risk factors for mental health problems among patients with polycystic ovary syndrome in Bangladesh: A nationwide cross—Sectional study. PloS One (2022) 17:e0270102. doi: 10.1371/journal.pone.0270102 35731829 PMC9216580

[7] Cooney LG Lee I Sammel MD Dokras A. High prevalence of moderate and severe depressive and anxiety symptoms in polycystic ovary syndrome: a systematic review and meta-analysis. Hum Reprod (2017) 32:1075–91. doi: 10.1093/humrep/dex044 28333286

[8] Santoro N. Polycystic ovary syndrome and mental health: a call to action. Fertility Sterility (2018) 109:799. doi: 10.1016/j.fertnstert.2018.02.121 29680313

[9] Douglas KM Fenton AJ Eggleston K Porter RJ. Rate of polycystic ovary syndrome in mental health disorders: a systematic review. Arch Women’s Ment Health (2022) 25:9–19. doi: 10.1007/s00737-021-01179-4 34499230

[10] Hoffmann MS Brunoni AR Stringaris A Viana MC Lotufo PA Benseñor IM. Common and specific aspects of anxiety and depression and the metabolic syndrome. J Psychiatr Res (2021) 137:117–25. doi: 10.1016/j.jpsychires.2021.02.052 33677215

[11] Berent-Spillson A Marsh C Persad C Randolph J Zubieta J-K Smith Y. Metabolic and hormone influences on emotion processing during menopause. Psychoneuroendocrinology (2017) 76:218–25. doi: 10.1016/j.psyneuen.2016.08.026 PMC5272799 27622993

[12] Kuang H Li Y Wu X Hou L Wu T Liu J. Acupuncture and clomiphene citrate for live birth in polycystic ovary syndrome: study design of a randomized controlled trial. Evidence-Based Complementary Altern Med (2013) 2013. doi: 10.1155/2013/527303 PMC3762180 24023577

[13] Wen J Chen X Yang Y Liu J Li E Liu J. Acupuncture medical therapy and its underlying mechanisms: a systematic review. Am J Chin Med (2021) 49:1–23. doi: 10.1142/S0192415X21500014 33371816

[14] Cao Y Chen H Zhao D Zhang L Yu X Zhou X. The efficacy of Tung’s acupuncture for sex hormones in polycystic ovary syndrome: A randomized controlled trial. Complementary Therapies Med (2019) 44:182–8. doi: 10.1016/j.ctim.2019.04.016 31126554

[15] Johansson J Redman L Veldhuis PP Sazonova A Labrie F Holm G. Acupuncture for ovulation induction in polycystic ovary syndrome: a randomized controlled trial. Am J Physiology-Endocrinology Metab (2013) 304:E934–43. doi: 10.1152/ajpendo.00039.2013 PMC4116535 23482444

[16] Wu X-K Stener-Victorin E Kuang H-Y Ma H-L Gao J-S Xie L-Z. Effect of acupuncture and clomiphene in Chinese women with polycystic ovary syndrome: a randomized clinical trial. JAMA (2017) 317:2502–14. doi: 10.1001/jama.2017.7217 PMC5815063 28655015

[17] Abdi H Abbasi-Parizad P Zhao B Ghayour-Mobarhan M Tavallaie S Rahsepar AA. Effects of auricular acupuncture on anthropometric, lipid profile, inflammatory, and immunologic markers: a randomized controlled trial study. J Altern Complementary Med (2012) 18:668–77. doi: 10.1089/acm.2011.0244 22788576

[18] Zhang X Qiu H Li C Cai P Qi F. The positive role of traditional Chinese medicine as an adjunctive therapy for cancer. Bioscience Trends (2021) 15:283–98. doi: 10.5582/bst.2021.01318 34421064

[19] Zhang B Shi H Cao S Xie L Ren P Wang J. Revealing the magic of acupuncture based on biological mechanisms: A literature review. BioScience Trends (2022) 16:73–90. doi: 10.5582/bst.2022.01039 35153276

[20] Wu L Li Y Yu P Li H Ma S Liu S. The application of acupuncture in obstetrics and gynecology: a bibliometric analysis based on Web of Science. Ann Palliative Med (2021) 10:3194–204. doi: 10.21037/apm-21-477 33849105

[21] Behboodi Moghadam Z Fereidooni B Saffari M Montazeri A. Measures of health-related quality of life in PCOS women: a systematic review. Int J Womens Health (2018) 10:397–408. doi: 10.2147/IJWH.S165794 30123008 PMC6078086

[22] Leung KF Liu FB Zhao L Fang JQ Chan K Lin LZ. Development and validation of the chinese quality of life instrument. Health Qual Life Outcomes (2005) 3:26. doi: 10.1186/1477-7525-3-26 15833138 PMC1090607

[23] Zhang H-L Huo Z-J Wang H-N Wang W Chang C-Q Shi L. Acupuncture ameliorates negative emotion in PCOS patients: a randomized controlled trial. Zhongguo Zhen jiu= Chinese Acupuncture & Moxibustion (2020) 40:385–90. doi: 10.13703/j.0255-2930.20191231-k0005 32275367

[24] Eshre TR A.-S.P.C.W. Group. Revised 2003 consensus on diagnostic criteria and long-term health risks related to polycystic ovary syndrome. Fertility sterility (2004) 81:19–25. doi: 10.1016/j.fertnstert.2003.10.004 14711538

[25] Chen Z Zhang Y Liu J Liang X Yu Q Qiao J. Diagnosis of polycystic ovary syndrome: standard and guideline of Ministry of Health of People’s Republic of China. Zhonghua Fu Chan Ke Za Zhi (2012) 47:74–5.

[26] Li R Qiao J Yang D Li S Lu S Wu X. Epidemiology of hirsutism among women of reproductive age in the community: a simplified scoring system. Eur J Obstetrics Gynecology Reprod Biol (2012) 163:165–9. doi: 10.1016/j.ejogrb.2012.03.023 22748844

[27] Zhao X Ni R Li L Mo Y Huang J Huang M. Defining hirsutism in Chinese women: a cross-sectional study. Fertility sterility (2011) 96:792–6. doi: 10.1016/j.fertnstert.2011.06.040 21762890

[28] Guyatt G Weaver B Cronin L Dooley JA Azziz R. Health-related quality of life in women with polycystic ovary syndrome, a self-administered questionnaire, was validated. J Clin Epidemiol (2004) 57:1279–87. doi: 10.1016/j.jclinepi.2003.10.018 15617954

[29] Stewart M. The Medical Outcomes Study 36-item short-form health survey (SF-36). Aust J Physiother (2007) 53:208. doi: 10.1016/S0004-9514(07)70033-8 17899676

[30] Leung K-f Liu F-b Zhao L Fang J-q Chan K Lin L-z. Development and validation of the chinese quality of life instrument. Health Qual Life Outcomes (2005) 3:1–19. doi: 10.1186/1477-7525-3-26 15833138 PMC1090607

[31] Zhao L Chan K Leung K Liu F Fang J. Application of factor analysis in development and validation of a new questionnaire for quality of life. Zhongguo Zhong xi yi jie he za zhi Zhongguo Zhongxiyi Jiehe Zazhi= Chin J Integrated Traditional Western Med (2004) 24:965–8. 15609589

[32] Chow YW Dorcas A Siu AM. The effects of qigong on reducing stress and anxiety and enhancing body–mind well-being. Mindfulness (2012) 3:51–9. doi: 10.1007/s12671-011-0080-3

[33] Jones G Benes K Clark T Denham R Holder M Haynes T. The Polycystic Ovary Syndrome Health-Related Quality of life questionnaire (PCOSQ): a validation. Hum Reprod (Oxford England) (2004) 19:371–7. doi: 10.1093/humrep/deh048 14747184

[34] Zheng R Qing P Han M Song J Hu M Ma H. The effect of acupuncture on glucose metabolism and lipid profiles in patients with PCOS: a systematic review and meta-analysis of randomized controlled trials. Evidence-Based Complementary Altern Med (2021) 2021. doi: 10.1155/2021/5555028 PMC8007365 33824676

[35] Dong H-x Wang Q Wang Z Wu X-k Cheng L Zhou Z-m. Impact of low frequency electro-acupuncture on glucose and lipid metabolism in unmarried PCOS women: a randomized controlled trial. Chin J Integr Med (2021) 27:737–43. doi: 10.1007/s11655-021-3482-z 34319506

[36] Lai M-H Ma H-X Yao H Liu H Song X-H Huang W-Y. Effect of abdominal acupuncture therapy on the endocrine and metabolism in obesity-type polycystic ovarian syndrome patients. Zhen ci yan jiu= Acupuncture Res (2010) 35:298–302. 21090334

[37] Chen J Xing H Li Q Li M Wang S. Regulative effects of the acupuncture on glucose and lipid metabolism disorder in the patients of metabolic syndrome. Zhongguo Zhen jiu= Chin Acupuncture Moxibustion (2017) 37:361–5. doi: 10.13703/j.0255-2930.2017.04.004 29231585

[38] Yu J Zhang Z Li C Zhang J Ding Z Zhu W. Serum lipid concentrations are associated with negative mental health outcomes in healthy women aged 35–49 years. Front Psychiatry (2021) 12:773338. doi: 10.3389/fpsyt.2021.773338 34795602 PMC8593134

[39] Huang R Fan J-G Shi J-P Mao Y-M Wang B-Y Zhao J-M. Health-related quality of life in Chinese population with non-alcoholic fatty liver disease: a national multicenter survey. Health Qual Life Outcomes (2021) 19:1–8. doi: 10.1186/s12955-021-01778-w 33962617 PMC8106221

[40] Soto-Angona Ó. Anmella G Valdés-Florido MJ De Uribe-Viloria N Carvalho AF Penninx BW. Non-alcoholic fatty liver disease (NAFLD) as a neglected metabolic companion of psychiatric disorders: common pathways and future approaches. BMC Med (2020) 18:1–14. doi: 10.1186/s12916-020-01713-8 32998725 PMC7528270

[41] Han J Guo X Meng X-J Zhang J Yamaguchi R Motoo Y. Acupuncture improved lipid metabolism by regulating intestinal absorption in mice. World J Gastroenterol (2020) 26:5118. doi: 10.3748/wjg.v26.i34.5118 32982113 PMC7495030

[42] Jung J Lee SM Lee M-J Ryu J-S Song J-H Lee J-E. Lipidomics reveals that acupuncture modulates the lipid metabolism and inflammatory interaction in a mouse model of depression. Brain Behavior Immun (2021) 94:424–36. doi: 10.1016/j.bbi.2021.02.003 33607237

[43] Wang L-H Huang W Wei D Ding D-G Liu Y-R Wang J-J. Mechanisms of acupuncture therapy for simple obesity: an evidence-based review of clinical and animal studies on simple obesity. Evidence-Based complementary Altern Med (2019) 2019. doi: 10.1155/2019/5796381 PMC6378065 30854010

[44] Gao H Tong X Hu W Wang Y Lee K Xu X. Three-dimensional visualization of electroacupuncture-induced activation of brown adipose tissue via sympathetic innervation in PCOS rats. Chin Med (2022) 17:48. doi: 10.1186/s13020-022-00603-w 35436959 PMC9016980

[45] Jung J Lee SM Lee MJ Ryu JS Song JH Lee JE. Lipidomics reveals that acupuncture modulates the lipid metabolism and inflammatory interaction in a mouse model of depression. Brain Behav Immun (2021) 94:424–36. doi: 10.1016/j.bbi.2021.02.003 33607237

[46] Gong Y Chang H Gao J-S Liu C-D Han B-W Wu X-K. Progress of researches on non-specific effect of acupuncture. Zhen ci yan jiu= Acupuncture Res (2019) 44:693–7. doi: 10.13702/j.1000-0607.180909 31532141

[47] Ho RS Wong CH Wu JC Wong SY Chung VC. Non-specific effects of acupuncture and sham acupuncture in clinical trials from the patient’s perspective: a systematic review of qualitative evidence. Acupuncture Med (2021) 39:3–19. doi: 10.1177/0964528420920299 32375500

[48] Yi G Yanjun Z Xiuyun W Tangping X Jinsheng C Chunxu Z. Study on the correlation between calcium ions and meridian acupoints. World Chin Med (2020) 15:970–5.

[49] Yang ES Li P-W Nilius B Li G. Ancient Chinese medicine and mechanistic evidence of acupuncture physiology. Pflügers Archiv-European J Physiol (2011) 462:645–53. doi: 10.1007/s00424-011-1017-3 PMC3192271 21870056

[50] Bajic Z Sobot T Skrbic R Stojiljkovic MP Ponorac N Matavulj A. Homocysteine, vitamins B6 and folic acid in experimental models of myocardial infarction and heart failure—How strong is that link? Biomolecules (2022) 12:536. doi: 10.3390/biom12040536 35454125 PMC9027107

[51] Wu X Li Z Sun W Zheng H. Homocysteine is an indicator of arterial stiffness in Chinese women with polycystic ovary syndrome. Endocrine connections (2021) 10:1073. doi: 10.1530/EC-21-0224 34355700 PMC8428028

[52] Wang J You D Wang H Yang Y Zhang D Lv J. Association between homocysteine and obesity: a meta-analysis. J Evidence-Based Med (2021) 14:208–17. doi: 10.1111/jebm.12412 33145936

[53] Chang H Xie L Ge H Wu Q Wen Y Zhang D. Effects of hyperhomocysteinaemia and metabolic syndrome on reproduction in women with polycystic ovary syndrome: a secondary analysis. Reprod biomedicine Online (2019) 38:990–8. doi: 10.1016/j.rbmo.2018.12.046 30979610

